# m^1^A Post-Transcriptional Modification in tRNAs

**DOI:** 10.3390/biom7010020

**Published:** 2017-02-21

**Authors:** Stephanie Oerum, Clément Dégut, Pierre Barraud, Carine Tisné

**Affiliations:** Institut de Biologie Physico-chimique (IBPC), CNRS, UMR 8261 CNRS/Université Paris Diderot, 13 rue Pierre et Marie Curie, Paris 75005, France; oerum@ibpc.fr (S.O.); c.degut@gmail.com (C.D.); pierre.barraud@cnrs.fr (P.B.)

**Keywords:** 1-methyladenosine, m^1^A, tRNA, methylation, TrmI, Trm6–Trm61, Trm10, Trmt10C

## Abstract

To date, about 90 post-transcriptional modifications have been reported in tRNA expanding their chemical and functional diversity. Methylation is the most frequent post-transcriptional tRNA modification that can occur on almost all nitrogen sites of the nucleobases, on the C5 atom of pyrimidines, on the C2 and C8 atoms of adenosine and, additionally, on the oxygen of the ribose 2′-OH. The methylation on the N1 atom of adenosine to form 1-methyladenosine (m^1^A) has been identified at nucleotide position 9, 14, 22, 57, and 58 in different tRNAs. In some cases, these modifications have been shown to increase tRNA structural stability and induce correct tRNA folding. This review provides an overview of the currently known m^1^A modifications, the different m^1^A modification sites, the biological role of each modification, and the enzyme responsible for each methylation in different species. The review further describes, in detail, two enzyme families responsible for formation of m^1^A at nucleotide position 9 and 58 in tRNA with a focus on the tRNA binding, m^1^A mechanism, protein domain organisation and overall structures.

## 1. Introduction

The biosynthesis and maturation of tRNA is a complex and multistep process that varies across organelles and organisms. Despite the variety, they still retain common features, such as transcription from a distinct transfer DNA (tDNA) gene, removal (processing) of the 5′-leader and 3′-trailer, intron splicing (where introns are present), addition of 3′-CCA for amino acid coupling, and several nucleotide modifications (reviewed in [1,2,3,4,5]). Post-transcriptional modifications of tRNAs, in which a large variety of modified nucleotides are enzymatically introduced onto the tRNA transcript [6,7], expand their chemical and functional diversity. In some cases, these modifications have been shown to increase the structural stability and induce correct folding of the tRNA [3,8,9,10]. The most frequent post-transcriptional modification of tRNA is methylation. Methylation occurs on almost all nitrogen sites of the nucleobases, and additionally on the oxygen of the ribose 2′-OH, C5 atom of pyrimidines, and C2 and C8 atoms of adenosine (reviewed in [11,12]). tRNA methylations are performed by a family of RNA methyltransferases (MTases) that comprises more than 60 members in humans. Based on sequence and topology comparisons, RNA MTases were classified into four superfamilies: Rossmann-fold MTases (RFM, class I of SAM-dependent MTases), SpoU–TrmD MTases (SPOUT, class IV of SAM-dependent MTases), radical-SAM, and FAD/NAD(P)-dependent MTases [11,13], the largest of which is the RFM family [14], followed by the SPOUT family [15,16]. MTases are further divided into tRNA MTase subfamilies named either ‘Trm’ or ‘Trmt’ followed by a number (e.g. Trm5) or a letter (e.g. TrmD), depending on the kingdom of origin for the enzyme.

In this review, we outline the known tRNA sites to undergo methylation on the N1 atom of adenosine to form 1-methyladenosine (m^1^A), and provide an overview of the proteins responsible for this modification at each position in the tRNA. We further discuss, in detail, the m^1^A modifications at the two best-characterized positions: 9 and 58.

## 2. m^1^A Modifications in tRNA

**Figure 1:**
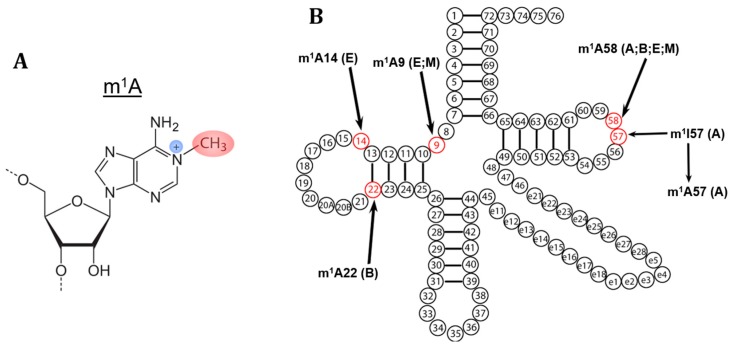
m^1^A modifications in tRNAs. (**A**) Chemical structure of the m^1^A modification on the adenosine base; (**B**) The m^1^A modification shown on a tRNA at all sites where the modification occurs (in red). The domain in which the modification has been identified is indicated as A: archaea, E: eukaryotes, and B: bacteria. Some modifications are found in mitochondria which is indicated as M.

In cytosolic (cyt) tRNA, the m^1^A modification (Figure 1A) occurs at five different positions (9, 14, 22, 57, and 58), two of which (9 and 58) are also found in mitochondrial (mt) tRNAs (Figure 1B and Table 1) [7]. The m^1^A modification in nucleotide position 14 (m^1^A14) is rare, and has so far only been identified in (cyt)tRNA^Phe^ from mammals [17,18], whereas the m^1^A22 modification has been identified only in tRNAs from bacteria [19,20,21,22]. The m^1^A57 modification was identified in archaea and exists only transiently as an intermediate leading to 1-methylinosine (m^1^I) by hydrolytic deamination [23,24]. The m^1^A58 modification occurs on (cyt)tRNAs from all three domains of life and further in (mt)tRNAs. The m^1^A9 modification is found in (cyt)tRNA from archaea or mammalian (mt)tRNAs [25,26].

## 3. The Biological Role of m^1^A Modifications in tRNA

The well-studied m^1^A9 and m^1^A58 modifications have both been linked to structural stability and/or correct folding of the tRNA. For the m^1^A9 modification, this link has been investigated in detail for human (mt)tRNA^Lys^, which exists in vitro as an extended hairpin structure [8,9] in equilibrium with the classical and functional tRNA ‘L-shape’ conformation [8,9]. The m^1^A9 modification shifts this equilibrium towards the ‘L-shape’ structure by disrupting a destabilizing Watson–Crick interaction that would otherwise form between A9 and U64 [9,25,27]. The impairment of the A9–U64 interaction allows U64 to bind to A50, allowing the tRNA to remain in the functional L-shaped structure [8,28]. Structural plasticity, similar to what is observed for (mt)tRNA^Lys^, was also reported for (mt)tRNA^Leu(UUR)^ and (mt)tRNA^Asp^, indicating that post-transcriptional modifications may also affect the fold of these (mt)tRNAs [29,30,31].

In *Drosophila*, small interfering RNA (siRNA)-mediated knockdown of the MTase performing the m^1^A9 modification was shown to be embryonically lethal [32]. In HeLa cells, the knockdown resulted in reduced mitochondrial respiration [33], indicating that this enzyme is also important for cell viability. The very rare autosomal recessive disorder combined oxidative phosphorylation deficiency 30 (COXPD30, MIM#616974) was recently attributed to missense mutations in a human mitochondrial m^1^A9 MTase [34,35,36]. Heterozygous or homozygous patients presented at birth with lactic acidosis, hypotonia, feeding difficulties, and deafness with death at five months of age after respiratory failure [36]. Analysis of patient fibroblasts showed decreased levels of the m^1^A9 MTase, indicative of lowered protein stability. In addition to the m^1^A9 activity, the affected MTase catalyses m^1^G9 formation [37] and is involved in 5′-end processing of mitochondrial precursor tRNA [37,38,39], making it difficult to attribute the patient phenotype to the m^1^A9 activity only.

**Figure 2:**
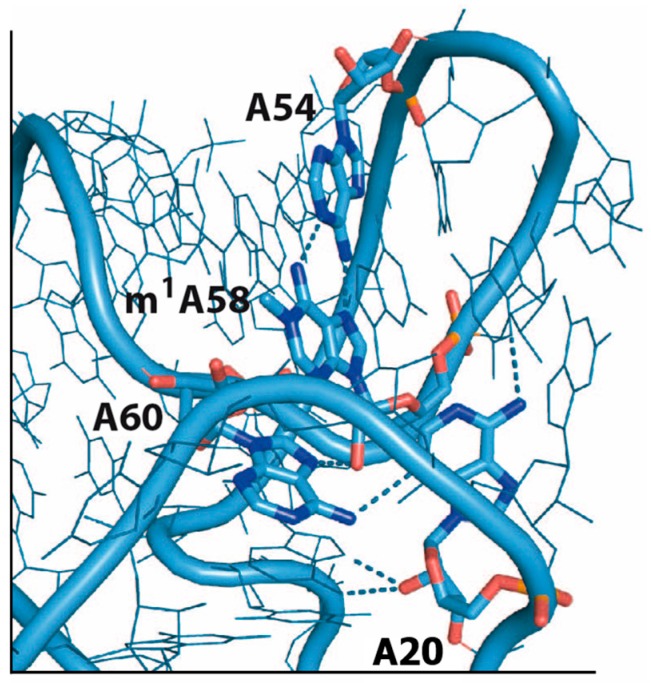
An overview of the ‘corner’ of the L-shaped initiator tRNA (tRNAi) from eukaryotes (PDB 1YFG). The nucleobases A20, A54, and A60 are highlighted along with the N1-modified A58 (m^1^A58). Hydrogen bonds are drawn as dotted lines.

The m^1^A58 modification has been related to structural thermostability of tRNA. The combination of m^1^A58 with two other post-transcriptional modifications (Gm18 and m^5^s^2^U54) was shown to increase the melting temperature of tRNAs from *Thermus thermophilus* by approximately 10 °C, compared to the unmodified transcript [10,40,41,42], and the lack of the enzyme forming m^1^A58 led to thermosensitivity in bacterial tRNAs [43]. The m^1^A58 in human tRNA^Lys^_3_ was shown to be crucial for reverse transcription fidelity and efficiency of retroviruses like HIV-1 [44]. m^1^A58 was also found to be important for maturation of the initiator tRNA^Met^ from yeast [45]. The initiator tRNA from eukaryotes (tRNAi) has a conserved A-rich T-loop (A54, A58, and A69), a conserved A20 and a shorter-than-average D-loop (seven nucleobases). These features cluster in the ‘corner’ of the L-shaped tRNA and the structure is maintained by a dense network of hydrogen bonds between the conserved adenines [46]. In this network, A58 forms hydrogen bonds to A54 and A60 (Figure 2) and the lack of m^1^A58 results in an abnormal tRNAi structure, guiding it for degradation. This might explain why deletion of the MTase N1-methylation A58 in yeast produces non-viable cells [47], and why exclusion of this MTase by siRNA-mediated knockdown gave rise to a slow-growth phenotype in human cells [48]. Recently, the human demethylase performing the reverse reaction (removal of the methyl group from the N1 atom of A58) was identified [49]. Knockdown of this enzyme resulted in elevated levels of tRNAi, agreeing with a role of m^1^A58 in tRNAi structure stability.

Little is known about the specific role of m^1^A14 and m^1^A22, but the MTase responsible for m^1^A22 in *Streptococcus pneumoniae* was shown to be essential for bacterium survival [50].

In addition to tRNA, the m^1^A modification was also identified on messenger RNA (mRNA) (reviewed in [51]). The modification was predominantly found in structured regions of the 5′ untranslated region, or close to canonical or alternative start codons [52,53], suggesting that also in mRNA this modification plays a role in RNA structural stability. The presence of the m^1^A modification on mRNA was linked to increased translation rates [52], and the level of the modification was shown to decrease in response to induced stress, i.e., glucose or amino acid starvation [52]. The DNA repair enzyme ALKBH3 that demethylates N1-methyldeoxyadenosine in single-stranded DNA [54,55] was suggested as a candidate for removing the methyl group from m^1^A in the mRNA [52].

## 4. Enzymes Responsible for the m^1^A Modifications in tRNA

**Figure 3:**
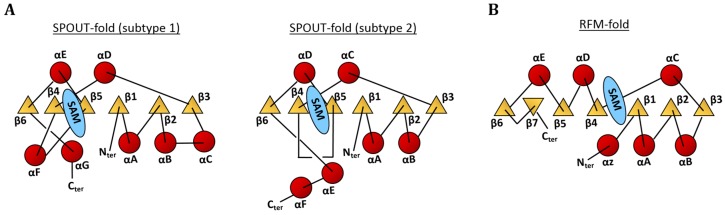
Topology diagrams of the classical folds for (**A**) the SPOUT superfamily (two subtypes), and (**B**) the Rossmann-fold methyltransferase (RFM) superfamily. SAM: *S*-adenosyl-l-methionine.

The MTases responsible for the m^1^A modification belong to either the RFM or the SPOUT superfamily of MTases, both of which utilise *S*-adenosyl-l-methionine (SAM) as the methyl donor, linking tRNA methylation to occurrence of ATP and methionine; the precursors for SAM catabolism. The SPOUT superfamily of proteins methylate bases and ribose moieties of tRNAs, but also rRNAs, in all three domains of life [11,15,16,56]. The SPOUT-fold comes in two subtypes, both resembling a Rossmann-fold in the first part and encompassing a trefoil knot in the second part (Figure 3A). The SPOUT domain is the catalytically active subunit of the MTases and binds the SAM cofactor. Many SPOUT MTases contain additional nucleic acid binding domains, indicating their primary function as MTases towards nucleic acids [16,57,58]. The RFM family contains RNA MTases and the majority of DNA MTases [11], and is named after the structural motif (Rossmann-fold) known for binding of adenosine-containing cofactors (SAM, NADH, etc.). The RFM-fold covers seven-stranded β-sheet flanked by six α-helices (Figure 3B). RFM MTases are often monomeric, although di-, tri-, and tetrameric oligomeric states have been reported. They often contain additional domains inserted throughout the RFM-fold, which play roles in substrate recognition [14]. The SPOUT and RFM MTases, responsible for m^1^A modifications at nucleotide position 9, 14, 22, and 58, are shown in Table 1, which further outlines the domain(s) of origin for each enzyme class and the cellular localisation of the substrate tRNA. The m^1^A9 and m^1^A58 MTases are well-studied and will be described in detail in later sections. The m^1^A22 MTase (TrmK) belongs to the COG2384 protein family and has orthologues in Gram-positive and Gram-negative bacteria, with no homologues identified in eukaryotes to date. TrmK is well conserved in the bacterial kingdom with enzymes from a number of pathogenic bacteria, e.g. *Vibrio cholerae*, *Listeria monocytogenes*, *Staphylococcus aureus*, *S. pneumoniae*, showing a high sequence identity (>40%). As highlighted in Table 1, the m^1^A14 MTase is yet unidentified, but an enzyme, partially purified from rat brain cortices, catalysed the reaction in vitro [59]. The gene encoding the protein is still unknown.

## 5. Mechanism for Formation of the N1-Methylation in tRNA

**Figure 4:**
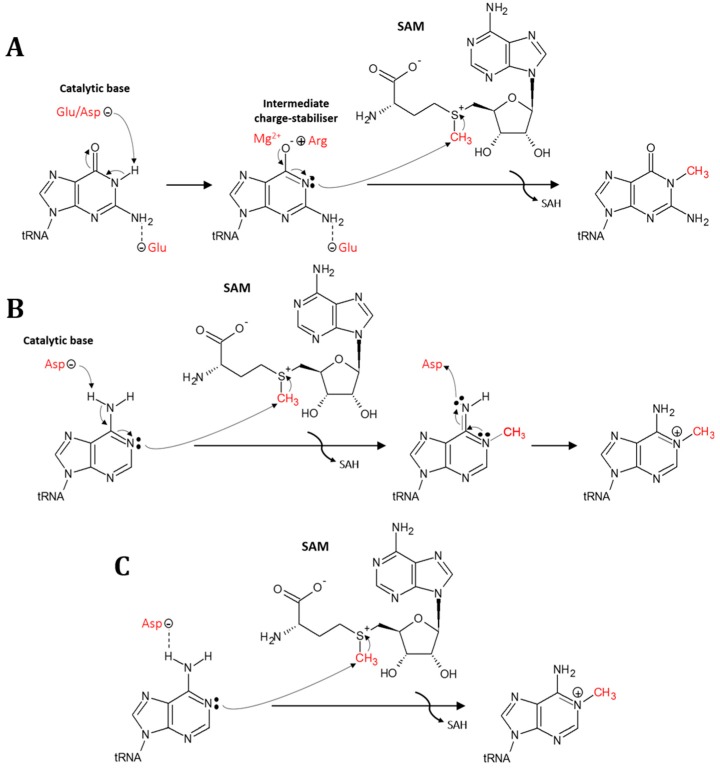
Mechanism for N1-methylation on purine bases using the cofactor *S*-adenosyl-l-methionine (SAM) converting it to *S*-adenosyl-l-homocysteine (SAH). (**A**) Mechanism for m^1^G37 formation by TrmD (with aspartate as catalytic base) or Trm5 (with glutamate as catalytic base). Only the guanine base of tRNA is depicted for clarity. (**B**) Suggested mechanism for m^1^A based on studies on TrmI (m^1^A58) with aspartate as the catalytic base. (**C**) Alternative mechanism for m^1^A based on studies on TrmI (m^1^A58) where aspartate serves merely to position the cofactor and methylation site for reaction.

N1-methylation can occur on both purines (adenine and guanine), with m^1^A occurring at a greater number of positions (9, 14, 22, 57, and 58) than m^1^G (9, 37) [7]. The mechanism by which the purines are methylated has been studied most extensively for guanine (m^1^G). Studies on the m^1^G37 specific SPOUT family member TrmD from the bacteria *Haemophilus influenzae* [60] and the m^1^G37 specific RFM family member Trm5 from the archaea *Methanocaldococcus jannaschii* [61,62] showed that the m^1^G37 mechanism involves a deprotonation of the N1 atom by an aspartate or glutamate residue (general base), a stabilisation of the resulting negative charge on the O6 atom by an arginine residue (intermediate charge-stabiliser), and an interaction with the N2 atom by a glutamate residue (Figure 4A). Subsequently, the activated N1 atom makes a nucleophilic attack on the reactive methyl group of SAM [63]. TrmD has recently been shown to further utilise a Mg^2+^ ion for stabilisation of the negatively-charged intermediate [64].

Due to the differences in protonation states of the N1 atom at physiological pH, the formation of m^1^A must proceed via a mechanism different from m^1^G. Two mechanisms for formation of m^1^A have been proposed, based on investigations on the m^1^A58 specific RFM family member TrmI from the eubacteria *T. thermophilus* [65,66]. In the first mechanism (Figure 4B), a deprotonation takes place as the initial step. In contrast to the N1 atom in guanosine, this atom in adenosine is not protonated at physiological pH, and the deprotonation by the Asp general base is, therefore, suggested to occur on the exocyclic N6 atom, resulting in the formation of an imino tautomer. This formation would activate the lone pair of the N1 atom for nucleophilic attack on the reactive methyl group of SAM, similar to the methylation of guanine [65]. In the second mechanism (Figure 4C), another role was suggested for the aspartate general base in the N1-methylation of adenine. Rather than deprotonating the N6 atom, it serves merely to position the methyl donor SAM and the target adenosine for methylation reaction [65]. A recent crystal structure of the human m^1^A58 MTase in complex with tRNA^Lys^_3_ [67] unfortunately did not provide information on the correct mechanism, as the position of A58 in the active site resembled a methylated nucleobase in a product-complex. Further studies are, thus, needed to fully elucidate the catalytic mechanism for m^1^A formation.

## 6. m^1^A58

The m^1^A58 MTases belong to the RFM superfamily and one of two subfamilies (Trm6 or Trm61). In eukaryotes, the m^1^A58 MTase located in the cytosol is composed of a catalytic protein unit from the Trm61 subfamily (Trm61A) and an RNA-binding protein unit from the Trm6 subfamily (Trm6) [45]. Sequence analysis has suggested that Trm6 and Trm61 share a common ancestor and arose via gene duplication and divergent evolution [68,69]. The mitochondrial m^1^A58 MTase consists of a single protein from the Trm61 family (Trmt61B), which is a paralogue to Trm61A from the cytosolic complex [70]. In archaea and bacteria, the m^1^A58 MTases belong to the TrmI subfamily and function without complex partners. These MTases were first identified as homologues of yeast Trm61 [43,71].

The eukaryotic complex of Trm6–Trm61 has been reported as a heterotetramer, whereas bacterial and archaeal TrmI proteins have been shown to form homotetramers [71]. Crystal structures are available for heterotetrameric Trm6–Trm61A complexes from *Saccharomyces cerevisiae* [72] and human (PDB 2B25). A structure of the human complex bound to tRNA is also reported [67]. Structures of homotetrameric TrmI proteins are available for the archaea *Pyrococcus abyssi* [73], and the bacteria *T. thermophilus* [65,66], *Aquifex aeolicus* [74], *Thermotoga maritima* (Joint Center for Structural Genomics 2003, PDB 1O54), and *Mycobacterium tuberculosis* [75].

### 6.1. Eukaryotes

**Figure 5:**
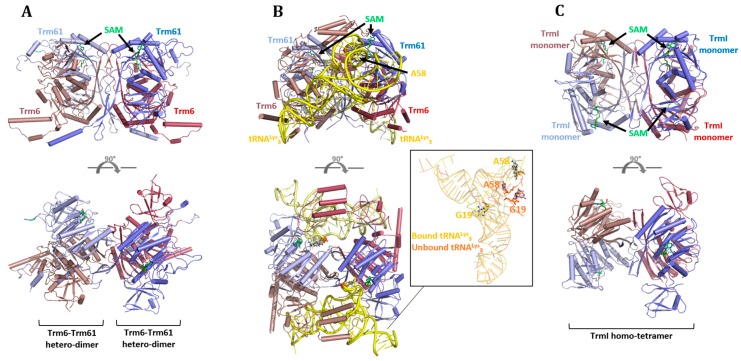
Structures of m^1^A58 tRNA MTases: (**A**) Trm6–Trm61 heterotetramer (PDB 5ERG); (**B**) Trm6–Trm61 heterotetramer bound to tRNA^Lys^_3_ (PDB 5CCB). The insert shows a superposition of free tRNA^Lys^_3_ (PDB 1FIR) and bound tRNA^Lys^_3_ (from PDB 5CCB). A58 and G19 are highlighted to emphasise the structural rearrangement of the tRNA upon binding; (**C**) TrmI homotetramer (PDB 2PWY).

In the complex of Trm6–Trm61 from *S. cerevisiae*, both subunits harbour an N-terminal domain linked to a C-terminal domain (Figure 5A) [72]. The C-terminal domains cover a Rossmann-fold and are very similar between the two subunits, whereas significant differences are found between the N-terminal domains. The N-terminal domain of Trm61 contains a short α-helix and three hairpin β-motifs, whereas Trm6 consists of a short α-helix with seven antiparallel β-strands and a highly flexible region with a number of positively-charged residues. Each subunit of the Trm6–Trm61 complex forms heterodimers that, again, assemble as a heterotetramer. The catalytic subunit of this complex (Trm61) binds the cofactor SAM; a binding that is made impossible in the other subunit (Trm6) by the loss of conserved motifs involved in accommodation of this cofactor. Each heterotetramer binds two tRNA molecules onto two distal, L-shaped surfaces on the protein complex (Figure 5B) [67]. tRNA undergoes large conformational changes during binding in which the D- and T-arm are separated (Figure 5B, insert). The T-loop contains the nucleobase to be modified (A58) and binds in the active site. The binding is stabilised by the formation of numerous hydrogen bonds with the C56 nucleobase and the sugar-phosphate backbone. A stabilising hydrogen bond is also formed between a phosphate O atom of C56 and a H atom of the exocyclic N6 atom of A58. No hydrogen bonds are observed between the protein complex and A58, and the orientation of this adenosine towards the bound *S*-adenosyl-l-homocysteine (SAH) resembles a methylated nucleobase. A conserved aspartate residue (Asp181) is found in close proximity to A58 (3.3 Å Asp181(Oδ1)-A58(N1)) and could serve as the catalytic base. In support of this, Asp181 was previously shown to be essential for m^1^A58 MTase activity in *T. thermophilus* [65]. The complex makes additional contacts with the tRNA substrate with binding of the acceptor stem to the N-terminal domain of the catalytic subunit Trm61, and binding of the T-stem/loop to an insert in the N-terminal domain of Trm6, not present in Trm61. The vast number of interactions with both complex subunits explain previous findings that both Trm6 and Trm61 are required for tRNA binding [76]. The interactions between tRNA and Trm6 help orient A58 for catalysis and may contribute to target specificity [67], providing a role for the non-catalytic subunit Trm6 in activity [45,77].

### 6.2. Archaea and Bacteria

In the homotetramer of TrmI proteins (Figure 5C), each molecule contains an N-terminal domain and a catalytic C-terminal domain [75], both of which are structurally highly conserved to Trm61 from the eukaryotic MTase [67]. The reported crystal structures of TrmI proteins from mesophilic, thermophilic, or hyperthermophilic organisms allowed a detailed analysis of the architecture of this protein family [78]. The analysis revealed a different molecular strategy for maintaining the tetrameric architecture and, thus, activity, under extreme conditions. One difference was observed for the type of intermolecular interactions utilised to maintain the tetrameric structure. For thermophilic bacteria, the structure is stabilised by a number of ionic interactions found across the dimer interfaces, whereas hyperthermophilic bacteria utilised a number of hydrophobic interactions. The tetrameric structure of most TrmI proteins is further stabilised by two bidentate ionic bonds that, in archaeal *P. abyssi*, were found to be replaced by four inter-subunit disulfide bridges.

No structure of a tRNA–protein complex with TrmI is currently available, but biochemical studies of *T. thermophilus* showed that each homotetramer accommodates up to two tRNA molecules [65]. Modelling the tRNA^Lys^_3_ from the human heterotetrameric complex onto TrmI from *T. thermophilus*, reveals that the substrate is likely to be accommodated similarly in the homotetrameric protein, suggesting that a comparable methylation mechanism is probably used by the two proteins.

### 6.3. Mitochondria

The m^1^A58 modification has been reported in a small number of (mt)tRNAs from humans and bovines [70,79,80]. The modification is performed by the MTase Trmt61B, shown to fully, or partly, methylate some (mt)tRNAs at A58 in vitro [70]. The functional role of m^1^A58 in these (mt)tRNAs remains to be established, but does not appear to affect processing, stability, or function of tRNA^Leu(UUR)^ [70]. Trmt61B forms a tetramer, presumed to resemble the homotetramers of TrmI proteins. In support of a similar structural arrangement between Trmt61B and TrmI, a phylogenetic analysis confirmed a bacterial origin of the human protein [70].

## 7. m^1^A9

**Figure 6:**
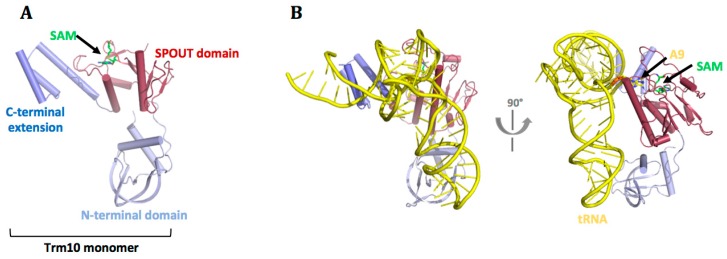
Structural data of Trm10. (**A**) Full-length Trm10 from *Sulfolobus acidocaldarius* (PDB 5A7Y). Residues 182–202 are not modelled. **(B)** Docking from [84] of *Escherichia coli* initiator tRNA (PDB: 3CW5) onto Trm10 from *S. acidocaldarius*.

The m^1^A9 MTases belong to the Trm10 subfamily of the SPOUT superfamily. In addition to the m^1^A9 modification, the Trm10 subfamily of MTases methylates guanosine in some organisms [11]. Currently, seven Trm10 proteins have confirmed N1-methylation activity for adenosine, guanosine, or both [37,81,82,83], of which four have been crystallised: human Trmt10A (PDB: 4FMW, Dong et al., 2012), *Schizosaccharomyces pombe* Trm10 (PDB: 4JWF) [83], *S. cerevisiae* Trm10 (PDB: 4JWJ) [83], and *Sulfolobus acidocaldarius* Trm10 (PDB: 5A7T) [84]. The Trm10 family of proteins contain a non-conserved N-terminal domain, a catalytic SPOUT domain with a subtype-2 SPOUT fold, and a non-conserved C-terminal extension. All crystal structures cover the SPOUT domain, but only the Trm10 MTase from *S. acidocaldarius* was reported as the full-length structure (Figure 6A). All crystal structures show monomeric protein domains, and this monomeric state was confirmed in studies of full-length Trm10 from yeast and archaea [83,84].

### 7.1. Eukaryotes

In eukaryotes, m^1^G9 is present in (cyt)- and (mt)tRNAs, whereas m^1^A9 is found only in animal (mt)tRNAs [26]. In humans, adenine occupies nucleotide position 9 in 14 out of the 22 (mt)tRNAs [26,85], all of which can be methylated on the N1 atom. m^1^A9 methylation on (mt)tRNAs is performed by Trmt10C, also known as mitochondrial ribonuclease P protein subunit 1 (MRPP1) [37]; a paralogue to the cytosolic m^1^G9 MTases Trmt10A and Trmt10B [37]. In contrast to its cytosolic paralogues, Trmt10C functions as a methyltransferase only when in complex with the unrelated mitochondrial metabolic enzyme 17β-hydroxysteroid dehydrogenase type 10 (HSD10) [39], belonging to the short-chain dehydrogenase/reductase (SDR) family of proteins [86,87,88]. In this complex, HSD10 is also known as mitochondrial ribonuclease P protein subunit 2 (MRPP2). The complex of Trmt10C–HSD10 (or MRPP1–MRPP2) catalyses the m^1^A9 formation (and m^1^G9) on tRNAs utilising SAM [89]. In vitro studies on the MTase activity of the Trmt10C–HSD10 complex revealed that both precursor and mature (mt)tRNA is a substrate for N1-methylation on guanosine and adenosine [37]. The role of HSD10 in this complex is unclear at present, but is likely independent of its catalytic function. In addition to the MTase activity, Trmt10C also interacts with the mitochondrial complement C1q binding protein (C1QBP) [38], and is involved in 5′-end processing of precursor tRNA as a component of the three-way (mt)RNase P complex [37,39].

The mechanism by which Trmt10C methylates the N1-atom in A9 is unclear, but—similarly to m^1^G9 formation—was shown to depend on an aspartate residue (Asp314) in the active site (putative catalytic base) [37] and a glutamine residue (Gln226) outside the immediate active site pocket [83]. Further studies are needed to elucidate how one enzyme can perform two such distinctively different mechanisms.

### 7.2. Archaea

**Figure 7:**
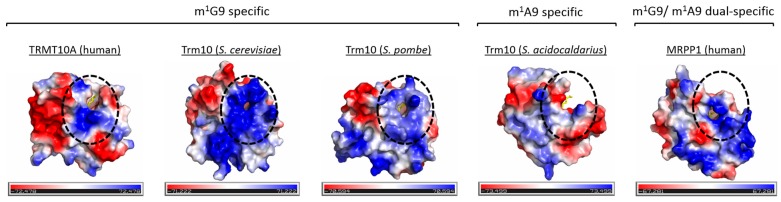
The electrostatic potential of the SPOUT domain for each Trm10 member for which a crystal structure is available. The SAM cofactor is shown as yellow sticks. The N1-methylation specificity is shown for each Trm10 member.

Nucleotide position 9 is the only position known to carry methylated adenosine and guanosine (m^1^A and m^1^G), where both reactions are sometimes even catalysed by the same enzyme. Trm10 proteins from *S. acidocaldarius* and *Thermococcus kodakarensis* have confirmed m^1^A9 activity [81]. Both of these proteins have an aspartate residue (Asp184 [84] and Asp206 [81], respectively), which is not present in Trm10 proteins from yeast harbouring only m^1^G9 activity. Mutation of this aspartate residue was shown to abolish m^1^A9 activity in the archaeal Trm10 proteins [84], and as this residue is not present in the m^1^G9-specific MTases from yeast, it was speculated to be one potential determinant for m^1^A activity. In human Trmt10C, this aspartate residue is replaced by leucine that, due to its uncharged nature, is likely to act differently in catalysis than a charged aspartate residue. Therefore, this amino acid position cannot be the sole determinant for m^1^A9 activity. Another aspartate residue is located deep in the active site pocket of human Trmt10C (Asp293), in a position corresponding to the Lys81 residue in the SPOUT protein TrmL from *Escherichia coli*, which was previously shown to be involved in catalysis but not tRNA binding [90]. Although the Asp293 residue could be involved in the MTase function in human Trmt10C, it seems unlikely to be the determinant for m^1^A9 activity, as this position is occupied by aspartate in the m^1^G9-specific Trm10 proteins from yeast, and is not conserved in m^1^A9 active Trm10 proteins from *S. acidocaldarius* and *T. kodakarensis*. Aside from the residues discussed above, alignment of the available Trm10 protein structures and their primary sequences show no other obvious amino acid candidates in the active site that could account for the differences between m^1^G9-specific (*S. cerevisiae* and *S. pombe*), m^1^A9-specific (*S. acidocaldarius*) and m^1^A9/m^1^G9 dual-specific (human Trmt10C and Trm10 from *T. kodakarensis*) Trm10 MTases. It is possible that the purine specificity could simply be due to differences in surface charge around the active site and size and/or layout of the purine-binding pocket (Figure 7), which could allow different Trm10 family members to accommodate different purine substrates, rather than to specific residues for catalysis. The active site pocket is more open for the m^1^G9-specific Trmt10A and m^1^A9-specific Trm10, compared to the other Trm10 proteins. However, no obvious similarities are observed within the m^1^G9-specific group of proteins that are also clearly different from the m^1^A9-specific Trm10, and altered in the m^1^G9/m^1^A9 dual-specific protein. A detailed understanding on how the substrate specificities are generated would benefit from a future high-resolution, substrate-bound crystal structure of Trm10 family members.

### 7.3. tRNA Recognition by Trm10 Proteins

Most SPOUT MTases are dimeric proteins, contrasting the monomeric nature of Trm10 proteins. Despite the differences in oligomeric state, a similar binding site is found for the SAM cofactor in the catalytic SPOUT domains. During catalysis, the dimeric SPOUT proteins involve residues from two catalytic SPOUT domains in binding of the purine to be modified [56]. As the SPOUT domain of Trm10 members are monomeric, the above active site organisation and tRNA binding is impossible, and the tRNA recognition for the Trm10 subfamily is, thus, hypothesised to be different from other SPOUT superfamily members. A different binding of tRNA is supported by findings that other SPOUT members need an intact dimer for tRNA binding [90]. These dimers still accommodate only one tRNA substrate [56,91,92], explained by conformational changes in the protein homodimer–tRNA complex that render the protein asymmetrical after tRNA binding [56]. No crystal structures are yet available for a Trm10 protein in complex with tRNA, but mutational studies on Trm10 proteins from yeast and archaea revealed that the non-conserved N-terminal domains directly interact with the tRNA substrate [83,84]. The assistance in tRNA binding by domains, additional to the enzymatic domain, has been shown for many SPOUT family members and other tRNA modifying enzymes [56,84,93,94,95]. A docking model for tRNA onto Trm10 from *S. acidocaldarius* showed that the N-terminal domain could interact with the acceptor stem of tRNA and that the C-terminal extension could be involved in remoulding the tRNA (Figure 6B), such that the otherwise buried adenosine-9 becomes surface-exposed for methylation [84]. Further studies are needed to fully elucidate the binding of tRNA to these monomeric SPOUT proteins.

## 8. Conclusions

The m^1^A modification occurs at five nucleotide positions in the tRNA (9, 14, 22, 57, and 58) and plays a number of biological roles, e.g., enhancing structural stability and inducing correct folding of the tRNA. The most well-studied m^1^A modifications are those occurring at nucleotide positions 9 and 58. The MTase responsible for the formation of m^1^A58 in eukaryotes is a heterotetrameric complex of two heterodimeric subunits (Trm6–Trm61) in cytosol, and a homodimer of one subunit (Trmt61B) in mitochondria. In archaea and bacteria, the m^1^A58 MTase is, likewise, a homotetramer of one subunit (TrmI). All so far characterised m^1^A9 MTases are monomers in solution as well as in their crystal structures. The monomeric arrangement of this subfamily of subtype-2 SPOUT proteins is unusual, as ‘regular’ subtype-2 SPOUT proteins are usually found as dimers. The monomeric nature of the Trm10 family can be explained by the presence of an additional helix (αF), sterically hindering otherwise subtype-2 SPOUT-like dimerization via helix-αE.

Binding of tRNA to the m^1^A9 and m^1^A58 MTase has been extensively studied, but so far a crystal structure exists only for the human Trm6–Trm61 complex bound to tRNA^Lys^_3_, in which both the catalytic and non-catalytic domain contribute to tRNA binding. Biochemical studies on the enzyme families of TrmI and Trm10 show that also these proteins utilise the non-catalytic domains for tRNA binding. In the crystal structure of human Trm6–Trm61, and the docking model of Trm10 from archaea, the non-catalytic domains interact with the conserved acceptor stem of tRNA. It is tempting to speculate that the role of these non-catalytic domains is to recognise a more universally conserved part of the tRNAs, if the immediate environment around the modified nucleobase shows low structural conservation.

The mechanism for formation for m^1^A has not yet been determined, but relies on a number of residues, e.g., aspartate and glutamine, in all families. Some Trm10 MTases catalyse solely m^1^A or m^1^G formation, whereas some are able to catalyse both. No studies have yet been able to explain the purine specificity with simple residue mutation studies and it might, thus, be due to differences in surface charge around the active site and size, and/or layout of the purine-binding pocket.

We believe that crystal structures of m^1^A MTases in complex with their tRNA substrates, and accompanying thorough studies on the m^1^A mechanism, would greatly benefit the field going forward. Furthermore, often a limited number of the same tRNAs are utilised across the field and we believe that the knowledge on tRNA modifications would benefit from a systematic mapping of modifications in identical nucleotide positions across all tRNAs both in vivo and in vitro.

## Figures and Tables

**Table 1:** Overview of the SPOUT and RFM methyltransferases (MTases) responsible for m^1^A modifications at nucleotide positions 9, 14, 22, and 58, with the domain(s) of origin for each enzyme class and the cellular localisation of the substrate tRNA.

tRNA nt-position	Domain (tRNA cellular location)	MTase Superfamily	MTase Subfamily
9	E (mt), A	SPOUT/class IV	Trm10 *
14	E (cyt)	Unknown	Unknown
22	B	RFM/class I	TrmK
58	E (cyt)	RFM/class I	Trm6/Trm61
58	E (mt)	RFM/class I	Trm61 *
58	A, B	RFM/class I	TrmI

nt: nucleotide, E: eukaryotes, A: archaea, B: bacteria, mt: mitochondria, cyt: cytosol. * Subfamilies are sometimes alternatively referred to as Trmt10 and Trmt61, respectively.
